# Gut Dysbiosis Driven by *CFTR* Gene Mutations in Cystic Fibrosis Patients: From Genetic Disruption to Multisystem Consequences and Microbiota Modulation

**DOI:** 10.3390/genes16091049

**Published:** 2025-09-06

**Authors:** Natalia Pawłowska, Magdalena Durda-Masny, Szczepan Cofta, Daria Springer, Anita Szwed

**Affiliations:** 1 Institute of Human Biology and Evolution, Faculty of Biology, Adam Mickiewicz University, 61-614 Poznan, Poland; natalia.pawlowska@amu.edu.pl (N.P.); mdurda@amu.edu.pl (M.D.-M.); 2 Department of Pulmonology, Allergology and Respiratory Oncology, Poznan University of Medical Sciences, 60-569 Poznan, Poland; scofta@ump.edu.pl (S.C.); daria.springer@usk.poznan.pl (D.S.)

**Keywords:** *CFTR* mutations, cystic fibrosis, gut microbiota, dysbiosis, intestinal biofilms, intestinal inflammation, CFTR protein modulators

## Abstract

Mutations in the *CFTR* genes causing cystic fibrosis (CF) are associated with the presence of thick, viscous mucus and the formation of biofilms in the gastrointestinal tract (GI) that impair intestinal homeostasis, triggering chronic inflammation, epithelial barrier dysfunction, and changes in the composition and activity of the gut microbiota. CFTR protein modulators represent a promising approach to enhancing lower GI function in patients with CF. The aim of the review is to present the complex relationships between the presence of *CFTR* gene mutations and the gut microbiota dysbiosis in patients with cystic fibrosis. Mutations in the *CFTR* gene, the molecular basis of cystic fibrosis (CF), disrupt epithelial ion transport and profoundly alter the gastrointestinal environment. Defective chloride and bicarbonate secretion leads to dehydration of the mucosal layer, increased mucus viscosity, and the formation of biofilms that favour microbial persistence, which together promote gut microbiota dysbiosis. This dysbiotic state contributes to impaired epithelial barrier function, chronic intestinal inflammation, and abnormal immune activation, thereby reinforcing disease progression. The interplay between CFTR dysfunction and microbial imbalance appears to be bidirectional, as dysbiosis may further exacerbate epithelial stress and inflammatory signalling. Therapeutic interventions with CFTR protein modulators offer the potential to partially restore epithelial physiology, improve mucus hydration, and foster a microbial milieu more consistent with intestinal homeostasis. The aim of this review is to elucidate the complex relationships between *CFTR* gene mutations and gut microbiota dysbiosis in patients with cystic fibrosis, with a particular emphasis on the clinical implications of these interactions and their potential to inform novel therapeutic strategies.

## 1. Introduction

Cystic fibrosis (CF) is classified among a group of autosomal recessive genetic disorders resulting from mutations in the *Cystic Fibrosis Transmembrane Conductance Regulator (CFTR)* gene, located on the long arm of chromosome 7 (7q31) [1]. The *CFTR* gene encodes the CFTR protein, an anion channel situated in the apical membrane of epithelial cells, primarily responsible for transporting chloride and bicarbonate ions across epithelial surfaces [2]. Proper ion transport is essential for maintaining the hydration and viscosity of mucus covering epithelial surfaces in organs such as the lungs, pancreas, and intestines. More than 2000 *CFTR* gene variants have been identified, with over 300 classified as pathogenic. These mutations are grouped into six (or sometimes seven) functional classes based on their molecular consequences. Class I mutations (e.g., G542X) result in no protein production due to premature stop codons. Class II mutations, such as the most common F508del, lead to misfolded proteins that are degraded before reaching the cell membrane. Class III mutations (e.g., G551D) produce proteins that reach the membrane but have defective channel gating. Class IV (e.g., R117H) and V mutations affect channel conductance or reduce protein synthesis, respectively, leading to milder phenotypes. Class VI mutations are associated with the increased turnover and instability of the CFTR protein at the membrane [1]. Regardless of the mutation class, impaired CFTR protein function disrupts the ion balance at epithelial surfaces, resulting in a reduced chloride and bicarbonate secretion and increased sodium absorption. This causes dehydration of the epithelial surface and leads to the formation and accumulation of thick, sticky mucus in many organs, primarily in the respiratory and gastrointestinal tracts. The accumulation of dehydrated mucus promotes chronic infections, inflammation, digestive disorders, and, consequently, malnutrition [3]. These changes, in turn, contribute to the overgrowth of pathogenic bacteria in the intestines, thereby exacerbating inflammation and placing additional strain on the organism. Consequently, a complex vicious cycle mechanism is established, which exacerbates the already compromised health of individuals with CF. Within the gastrointestinal tract, the presence of thick mucus has been demonstrated to disrupt the balance of the intestinal microbiota, reducing the abundance of beneficial commensal bacteria and promoting colonisation by opportunistic pathogens. The development of chronic intestinal inflammation leads to impaired nutrient absorption, which can consequently cause or aggravate malnutrition. Malnutrition, in turn, weakens the immune system function and increases the frequency of infections [4]. Moreover, the frequent use of antibiotics to treat infections in CF patients further exacerbates intestinal dysbiosis, reinforcing the cycle of gut inflammation and microbial imbalance [5,6,7,8]. The aim of our review is to present the complex relationships between the presence of *CFTR* gene mutations and the composition and activity of the gut microbiota in patients with cystic fibrosis. We explore how mutations in the *CFTR* gene at the molecular level contribute not only to ion transport dysfunction but also to downstream effects on intestinal homeostasis, including microbiota imbalance, chronic inflammation, and epithelial barrier disruption. Special emphasis is placed on the interplay between genetic defects and microbial alterations in the gastrointestinal tract as a contributing factor to disease progression in CF.

## 2. The Composition of the Gut Microbiota of Adults with the *CFTR* Gene Mutation Is Dominated by Pathogenic Bacteria and a Reduced Amount of Short-Chain Fatty Acid Producers, in Comparison to Healthy Individuals

The human gut microbiota is a complex ecosystem comprising various types of bacteria, as well as viruses, fungi, and archaea. These microorganisms play a pivotal role in maintaining bodily homeostasis [8]. Their presence is essential for a variety of biological processes, including metabolism, digestion, nutrient absorption, and immune modulation. The gut microbiota of healthy individuals is characterised by high diversity and richness. The most prevalent types of bacteria that constitute a healthy gut microbiota include *Bifidobacterium*, *Lactobacillus*, *Faecalibacterium*, *Roseburia*, and *Bacteroides* [9]. *Bifidobacterium* and *Roseburia* are responsible for the fermentation of dietary fibre and the production of short-chain fatty acids (SCFAs) [10]. SCFAs help regulate inflammatory processes (by inhibiting intestinal inflammation), enhance the absorption of nutrients, minerals, and vitamins, and contribute to the regulation of glucose levels [11]. Fibre fermentation products serve as substrates for other bacteria, such as *Faecalibacterium*, which also participate in SCFA production. These bacteria produce bioactive metabolites that provide protective effects, stabilise the microbiota environment, and support intestinal microbial balance [12]. Notably, they produce butyrate, a key metabolite for intestinal epithelial health, due to its anti-inflammatory properties and ability to promote epithelial regeneration [13]. Furthermore, the enzymes glucuronidase and xylanase can break down dietary polysaccharides into oligosaccharides, which serve as an excellent substrate for a genus of bacteria, such as *Roseburia* [14]. Consequently, intestinal colonisation by *Roseburia* is promoted [14]. Another important group, *Lactobacillus*, has been identified as a key player in the conversion of carbohydrates into lactic acid through fermentation [15]. These bacteria produce bacteriocins that create conditions favourable to the growth of beneficial microorganisms such as *Bifidobacterium* [16]. The presence of *Bifidobacterium* is supported, among other mechanisms, by co-feeding interactions with bacteria of the genus *Bacteroides*. *Bacteroides* can degrade complex plant polysaccharides into simpler oligosaccharides, which then serve as a substrate for fermentation by *Bifidobacterium* [17]. Certain *Bacteroides* species produce polysaccharide A, a substance that induces regulatory T cells in the intestines and thereby exerts immunomodulatory and inflammatory response-inhibiting effects [18]. Moreover, some species support the development and function of the mucosal barrier, protecting the intestines from pathogens [19].

A comparison of the gut microbiota of adults with *CFTR* gene mutations and that of healthy individuals reveals an increase in pathogenic and opportunistic bacteria, including *Escherichia coli*, *Pseudomonas aeruginosa*, *Staphylococcus aureus*, and *Clostridium difficile* [20] [Table 1]. *E. coli* and *P. aeruginosa* employ distinct adaptive mechanisms [21]. These organisms compete for the availability of resources, primarily nitrogen, which may be limited in the intestines. Toxins produced by *E. coli* disrupt nutrient absorption in humans, leading to gut inflammation [22]. Both *E. coli* and *P. aeruginosa* possess an efflux pump mechanism that enables them to excrete toxic substances, including antibiotics, heavy metals, and toxins produced by other bacteria. This function is essential for survival in the harsh environment of the gastrointestinal tract [23]. *E. coli* also responds to membrane phospholipid breakdown by releasing ethanolamine, which can be further metabolised by other bacteria [24]. Ethanolamine, a nitrogen-rich molecule, acts as a signalling factor in the gut microbiome, promoting the growth of pathogens, such as *P. aeruginosa*. *P. aeruginosa* has been shown to disrupt the gut microbial balance, thereby facilitating its own dominance [25]. Because the digestive and absorptive processes in the intestines leave nitrogen residues, including ammonia, which are present in a free form or as amino acids. These amino acids can be used by bacteria for protein synthesis [26]. However, under conditions of a low dietary protein intake or after digestion is complete, nitrogen availability can be significantly reduced [27]. In response, bacteria such as *E. coli* and *P. aeruginosa* adapt by utilising alternative nitrogen sources, such as ethylamine, enabling them to survive and maintain metabolic activity in the gut [26]. Another bacterium frequently encountered in the gastrointestinal tract of individuals with a disease is *S. aureus*. This bacterium employs an iron-regulated surface determinant (Isd) system, which enables it to compete for available iron [28]. However, *P. aeruginosa* pyoverdine competes effectively with the Isd system of *S. aureus*, reducing its iron acquisition capacity and limiting its growth. *S. aureus* has been observed to release peptides from casein, the major milk protein [29]. *P. aeruginosa* can use these peptides in the process of gluconeogenesis, converting them into sugars and other energy compounds [30]. This process facilitates the reciprocal development of both bacteria: *P. aeruginosa* acquires an additional carbon source, while *S. aureus* benefits from metabolites secreted by *P. aeruginosa*, enhancing its survival and enabling its coexistence in environments where other bacteria may not thrive [30]. A final bacterium exerting a significant influence on the gut microbiota composition is *C. difficile* [31]. *C. difficile* is responsible for the production of two major toxins, A and B, which exert potent cytotoxic effects on intestinal epithelial cells. These toxins modify key regulators of the actin cytoskeleton, disrupting cell shape and integrity. Consequently, epithelial cells undergo a programmed cell death, compromising the barrier function and facilitating invasion [32]. Short-chain fatty acids (SCFAs) reduce the intestinal pH to levels that decrease the *C. difficile* toxin’s activity, thereby protecting the epithelium and inhibiting the growth of pathogens. Both *C. difficile* and SCFA-producing bacteria compete for undigested polysaccharides in the large intestine. *C. difficile* rapidly imports and metabolises these carbohydrates for energy, fuelling its own growth and toxin production [33]. Conversely, SCFA producers convert the same substrates into acids, depleting nutrients and acidifying the environment, thereby further inhibiting the *C. difficile* virulence [34]. Thus, competition for resources and environmental modification are pivotal factors in determining whether *C. difficile* causes infection or is suppressed by the resident microbiota [35].

## 3. The Presence of Pathogenic Bacteria in the Intestines Leads to Changes in the Composition of Gastrointestinal Mucus in Adults with *CFTR* Gene Mutations

Mucus fulfils a fundamental role in mammals, protecting various body systems, including the respiratory and digestive systems. Its structure enables it to act as a dynamic barrier, separating the microbiota from epithelial cells while allowing the exchange of nutrients and metabolic signals. Its primary function is to protect mucosal surfaces from pathogens, toxins, and irritants while maintaining a balanced microbial environment [36]. Mucus fulfils a dual role as both a physical and biochemical barrier, preventing the entry of potentially harmful agents into the body. Its composition—including water, mucins, electrolytes, and antimicrobial factors—renders it a highly effective first line of defence against infections. Within the gastrointestinal tract, mucus plays a vital role in protecting the intestinal mucosa and regulating interactions with the microbiota [37]. In healthy individuals, mucus is characterised by adequate hydration, which is essential for the optimal viscosity required for effective mucociliary clearance in the respiratory tract and unobstructed transport in the gastrointestinal tract. Adequately hydrated mucus supports digestion and nutrient absorption while also acting as a barrier to toxins and pathogens [38]. Importantly, mucus functions not only as a physical barrier but also as a dynamic interface through which the microbial community exerts its influence. The composition of the gut microbiota has a direct impact on mucus structure and function [39]. The beneficial microbes, including *Lactobacillus* and *Bifidobacterium*, adapt to this environment by binding tightly to the mucus layer [40]. These bacteria stabilise the mucus architecture, compete with pathogens for binding sites, and enhance mucus immunity. They also stimulate goblet cells to ensure adequate mucin production, thereby maintaining the protective properties of mucus. Conversely, dysbiosis of the gut microbiota can disrupt this interaction [41]. Pathogenic bacteria, including *E. coli* and *C. difficile*, produce enzymes that degrade mucins, leading to thinning of the mucus layer and weakening of the epithelial barrier [42]. This degradation increases pathogen and toxin access to the epithelium, promoting inflammation and further compromising barrier integrity [43]. The loss of commensals removes their competitive and regulatory effects, while also reducing the signals required for proper mucin turnover and secretion [36]. In the gastrointestinal tract of patients with CF, thick mucus forms a mechanical barrier to digestive enzymes, particularly pancreatic lipase, resulting in the impaired digestion and absorption of fat. The resultant effect of this is poor nutrient absorption, steatorrhea (i.e., the presence of fatty stools), and an increased risk of malnutrition. Thick mucus also impedes the diffusion of digested nutrients into the microvilli, hindering glucose, amino acid, and fatty acid absorption [44]. The proximity of digested nutrients to the absorptive surface of the intestinal epithelium is crucial for efficient absorption; excess mucus disrupts this proximity. Moreover, the composition of mucus in CF is altered, promoting intestinal dysbiosis [45]. Excessive mucin production, combined with impaired clearance, favours the colonisation of opportunistic pathogens such as *P. aeruginosa*, *S. aureus*, or *C. difficile*. These bacteria can utilise mucins as a carbon source, producing enzymes that further degrade the mucus barrier [42]. Mucin breakdown products, in conjunction with components from lysed host cells, function as growth substrates for these microorganisms, enhancing colonisation. The density and viscosity of mucus hinder its clearance, facilitating the adhesion of pathogens such as *P. aeruginosa* to the epithelium [36]. Consequently, mucus loses its protective properties, perpetuating a cycle of inflammation and infection.

## 4. Biofilms Are a Consequence of Dysbiosis of the Intestinal Microbiota and Bacterial Defence Mechanisms in *CFTR* Gene Mutations

Biofilms are defined as three-dimensional structures composed of bacteria embedded in an extracellular polymeric substance (EPS) matrix and secreted products [45]. Biofilm formation is a dynamic, multistage process. The biofilm confers protection against antibiotics and immune cells, thereby exacerbating chronic inflammation and causing tissue damage. Its extracellular matrix forms a physical barrier that impedes the penetration of antibodies and other immune components [46]. Bacteria within biofilms exist in diverse metabolic states. The presence of biofilms in the gastrointestinal tract have been shown to play a significant role in disorders affecting digestion and nutrient absorption [47]. The colonisation of the intestinal epithelium by biofilms impedes digestive enzyme and nutrient access to enterocytes, reducing the efficiency of both digestion and absorption [48]. The intricate architecture of the biofilm hinders the activity of digestive enzymes, including amylase and lipase, limiting macromolecule breakdown and reducing nutrient availability [49].

Pathogenic bacteria, including *P. aeruginosa*, *S. aureus*, and *E. coli*, can significantly modify the structure and protective function of intestinal mucus [50]. Dense surface communities formed by these organisms physically block digestive enzymes such as amylase and lipase from reaching nutrients. In addition, they secrete toxins and proteolytic enzymes that directly weaken the gel matrix. As virulence factors degrade mucin glycoproteins, the mucus layer becomes thinner, less cohesive, and more permeable, compromising its barrier function. Concurrently, these pathogens release inflammatory signals that stimulate enterocyte apoptosis and disrupt epithelial tight junctions [51]. The resulting increase in intestinal permeability facilitates bacterial translocation and the spread of their products to the submucosa and bloodstream, driving local inflammation and systemic immune activation. As inflammation intensifies, goblet cells may alter mucin production, often producing atypical mucus that is either excessively viscous (trapping microbes in pockets) or abnormally loose (providing incomplete coverage). Concurrently, beneficial commensals such as *Bifidobacterium* and *Lactobacillus* encounter difficulties in maintaining their position. Under normal conditions, these species bind to the mucus scaffold, outcompete pathogens for binding sites, and secrete signals that promote healthy goblet cell activity and balanced mucin glycosylation. In pathogen-dominated environments, however, they shift towards enhanced carbohydrate consumption and metabolite production, which acidifies the microenvironment and creates a pH gradient unfavourable to commensal microbes. The loss of these protective microbes further accelerates mucin degradation and barrier breakdown [45,46,47,48,49,50,51].

**Figure 1:**
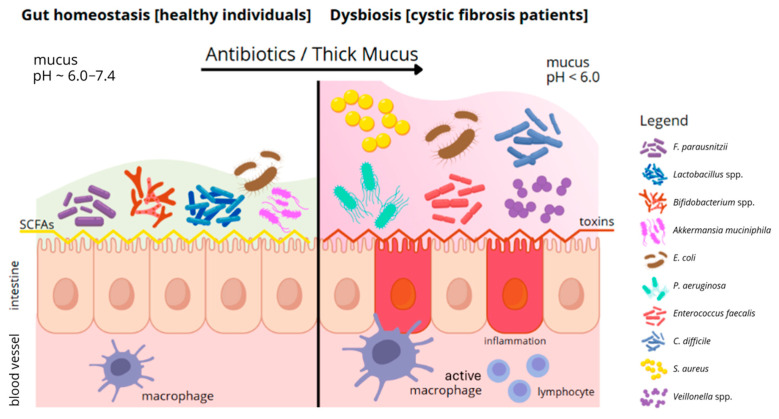
Schematic comparison of gut biofilm-forming microbial populations in healthy individuals (**left**) and cystic fibrosis patients (**right**), highlighting host–microbe interactions at the molecular level. The figure was created using Canva (www.canva.com, accessed on 31 August 2025).

Figure 1 illustrates how *CFTR* gene mutations indirectly shape the gut microbial landscape. In individuals with cystic fibrosis, mutations in the *CFTR* gene impair ion transport and mucus hydration, thereby altering the intestinal microenvironment at the epithelial surface. This creates selective pressure that favours biofilm-forming pathogenic taxa (*P. aeruginosa*, *C. difficile*, and *S. aureus*), while reducing colonisation by beneficial commensals (*Lactobacillus*, *Bifidobacterium* and *Faecalibacterium prausnitzii*). The resulting dysbiosis is further aggravated by antibiotic-driven shifts in microbial gene expression and horizontal gene transfer. Together, these microbial and host genetic factors synergistically drive chronic intestinal inflammation, barrier dysfunction, and disrupted immune–microbiota crosstalk.

## 5. The Function of the Immune System Is Closely Linked to the Composition of the Gut Microbiome

The immune system plays a pivotal role in defending against infection and injury; however, in individuals with CF, its function is significantly compromised [52]. Chronic inflammation, recurrent bacterial infections, and epithelial damage lead to a sustained immune activation and ultimately immune exhaustion [53]. Persistent bacterial infections necessitate extensive antibiotic therapy, which in turn reduces gut microbial diversity and weakens systemic immunity [54]. In a healthy gut, *Lactobacillus* communities predominate, with *Bifidobacteria* also present. These bacteria produce metabolites that fortify epithelial barriers and regulate immune cells to ensure balanced responses [55]. SCFAs, generated through bacterial fibre fermentation, exert multiple anti-inflammatory effects, including the suppression of pro-inflammatory cytokines, promotion of regulatory T cell differentiation, and enhancement of the tight junction and mucin production, thereby limiting systemic inflammation [56].

The microbiota of CF patients is characterised by a loss of key commensals that produce anti-inflammatory and SCFA metabolites. This shift promotes the proliferation of pro-inflammatory species, such as *E. coli* and *E. faecalis* [57]. Reduced SCFAs levels weaken the protective mucus layer of the gastrointestinal tract, enabling the passage of harmful bacterial products through the epithelium. This stimulates the immune cells to secrete cytokines that trigger inflammation [58]. Furthermore, the viscoelastic properties of CF mucus alter biofilm dynamics, allowing pathogens such as *P. aeruginosa* and *S. aureus* to adhere more firmly, form dense microcolonies, and resist clearance [59,60]. Within biofilms, bacteria secrete proteases and inflammatory mediators that disrupt the mucosal barrier and enhance local inflammation. At the same time, mucus entrapment restricts antibiotic penetration and immune cell access [61]. Gut microbial diversity is further reduced, with decreases in *Faecalibacterium* and *Roseburia* and increases in *E. coli* and *E. faecalis*, both associated with inflammation [62]. Lower SCFA-producing bacteria in CF patients diminish acetate, propionate, and butyrate levels, further weakening the mucosal barrier and facilitating bacterial translocation into the lamina propria. Once across the epithelial barrier, lipopolysaccharide and other microbial molecules activate Toll-like receptor signalling on immune cells, driving pro-inflammatory cytokines production. This cascade is responsible for the low-grade intestinal inflammation characteristic of the CF gut and correlates with symptom severity and malnutrition risk. Circulating microbial products may also exacerbate inflammation in distant organs, contributing to the gut–lung axis of CF, whereby gut dysbiosis amplifies lung immune system activation [63].

Several factors contribute to this dysbiotic profile in CF: frequent antibiotic therapy, high-fat diets tailored to patients’ nutritional needs, and direct consequences of CFTR protein dysfunction on the gut environment. Antibiotics, although essential for controlling chronic respiratory infections, also deplete commensal populations and select for resistant, pro-inflammatory strains, thereby worsening the imbalance and barrier disruption [20]. SCFA deficiency impairs the tight junction and mucin production, compromising epithelial integrity [64]. Furthermore, this deficiency disrupts immune regulation by reducing signals that normally promote T regulatory cell differentiation and IL-10 secretion. In such an environment, opportunistic pathogens colonise mucosal surfaces, releasing inflammatory mediators and proteases that further weaken host defences [65]. Restoring a balanced microbiota through dietary prebiotics, probiotic supplementation, or therapies that directly increase SCFA levels represents a promising strategy for improving barrier function and reducing chronic inflammation in individuals suffering from cystic fibrosis. The combined effects of gut dysbiosis, SCFA deficiency, and mucus-associated biofilms perpetuate a vicious cycle of barrier breakdown, microbial invasion, and chronic inflammation [66]. Strategies to restore microbial balance—including prebiotic fibres, targeted probiotics, and CFTR protein modulators that indirectly rebalance the gut ecosystem—have reduced gut inflammatory markers and improve barrier function in preliminary studies [67]. A comprehensive therapeutic approach should therefore address both microbial composition and mucus pathology to ameliorate inflammation and improve outcomes in CF.

## 6. CFTR Protein Modulators Make the Gut Microbiome of Cystic Fibrosis Patients Resemble a Healthy Gut Microbiome

**Figure 2:**
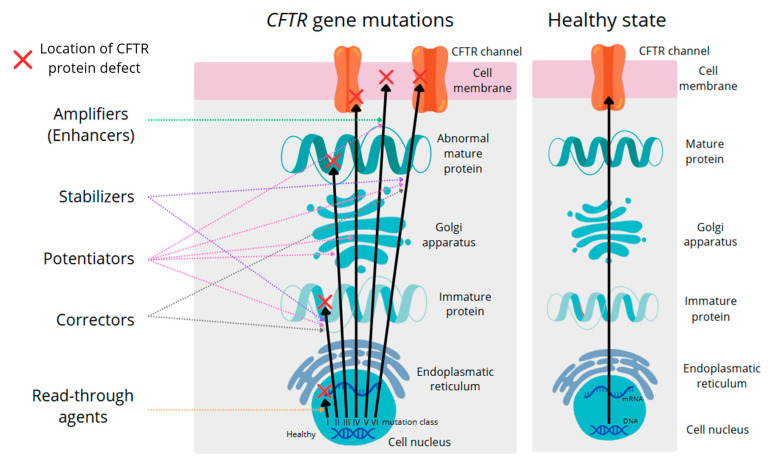
Mechanism of action of CFTR modulators in relation to specific *CFTR* gene mutation classes, compared to normal *CFTR* gene expression and protein function. The figure illustrates how various classes of CFTR protein modulators—including potentiators, correctors, stabilisers, read-through agents, and amplifiers—target specific molecular defects caused by different classes of *CFTR* gene mutations. Each modulator class is designed to address distinct abnormalities in *CFTR* gene transcription, mRNA processing, translation, protein folding, intracellular trafficking, channel gating, or membrane stability, thereby partially restoring CFTR protein function in individuals with cystic fibrosis. The figure was created using Canva (www.canva.com, accessed on 31 August 2025).

CFTR protein modulator therapy has transformed the landscape of CF care by targeting the underlying defect in CFTR protein processing and function, rather than merely addressing downstream consequences [68]. CFTR protein modulators can be categorised into five distinct classes: potentiators, correctors, enhancers, read-through factors, and stabilisers [Figure 2]. Each of these modulators have been designed to target a specific defect in the production or function of the CFTR protein. The first-class potentiators enhance the activity of CFTR channels that reach the cell surface. Ivacaftor, approved by the FDA on 31st January 2012, for patients aged six years and older with the G551D gating mutation, was the first potentiator and demonstrated significant improvements in lung function, sweat chloride levels, and nutritional status. Its indication was later expanded to cover more than two dozen additional gating and conductance mutations. More recently, novel potentiators have been developed to extend these benefits to less common *CFTR* variants. Correctors enable misfolded CFTR proteins to escape degradation within the endoplasmic reticulum and reach the cell membrane. The first approved combination of correctors and potentiators, lumacaftor–ivacaftor (Orkambi), was authorised in July 2015 for patients homozygous for the F508del mutation. Although improvements in lung function were less pronounced than with ivacaftor alone, Orkambi significantly reduced pulmonary exacerbations and an improved body mass index. In February 2018, tezacaftor–ivacaftor (Symdeko) showed similar benefits with fewer drug interactions and adverse effects. The most advanced therapy to date, the triple combination elexacaftor–tezacaftor–ivacaftor (Trikafta), approved in October 2019, further stabilises the CFTR structure, improves folding, and enhances channel gating. This treatment benefits approximately 90% of patients with CF, including those carrying one or two copies of the F508del mutation, leading to an average improvement in lung function of 10 to 14 percentage points, in addition to substantial reductions in sweat chloride levels and fewer pulmonary exacerbations. In addition, enhancers and read-through agents offer strategies for patients whose *CFTR* mutations reduce protein levels or introduce premature stop codons. Enhancers such as nesolicaftor increase *CFTR* mRNA stability and transcription, thereby expanding the pool of protein available for correction, which is relevant for class V mutations. Read-through agents, such as ataluren, allow ribosomes to bypass nonsense mutations, enabling the synthesis of nearly full-length CFTR proteins. Although still experimental, these therapies provide hope for individuals with truncating variants. The most recent class, stabilisers, has been designed to prolong the lifespan of properly folded CFTR at the plasma membrane, thereby increasing channel durability and potentially improving the efficacy of other modulators. Collectively, these five classes constitute a precision medicine toolkit that restores the CFTR function at multiple stages, including production, assembly, transport, gating, and longevity. Beyond respiratory benefits, CFTR modulators correct ion and water transport in the gastrointestinal tract, resulting in more hydrated mucus, reduced gut inflammation, a healthier microbiota, and improved nutrient absorption. In particular, triple combination regimens, specifically elexacaftor/tezacaftor/ivacaftor, have been shown to normalise proximal gut pH, improve mucus hydration, and decrease intestinal inflammation. These changes promote the growth of beneficial SCFA-producing taxa, reduce dysbiosis, and limit the overgrowth of pro-inflammatory organisms. Emerging evidence indicates that modulators can lower intestinal inflammatory markers such as calprotectin and pyruvate M2 kinase in stool samples, suggesting reduced gut inflammation. Improvements in microbial balance enhance nutrient absorption, contribute to the weight gain and may also mitigate systemic inflammation via the gut–lung axis. However, the efficacy of modulators is contingent on specific *CFTR* genotypes, leaving approximately 10 percent of patients with rare or complex mutations without a targeted treatment. This highlights the ongoing need for novel drug development and personalised approaches. Recent studies have shown that CFTR modulators, particularly ETI, are associated with increased gut microbiota diversity in individuals diagnosed with cystic fibrosis [69,70,71]. This modulation of the gut microbiota likely contributes to improved gastrointestinal health and overall well-being in patients with CF.

*CFTR* gene modulators have been shown to exert beneficial effects in individuals with cystic fibrosis. This is evidenced by their ability to enhance mucus hydration and reduce biofilm formation, thereby contributing to the restoration of a healthier gut microbiota. These positive changes in microbiome shifts include an increased abundance of health-promoting taxa and a reduction in opportunistic pathogens. Such changes have been demonstrated to improve gut health, nutrient absorption, and systemic metabolic outcomes, including weight gain and improved immune regulation.

## 7. Conclusions

CF is characterised by the production of thick mucus and the formation of biofilms in the gastrointestinal tract. These phenomena disrupt the normal microbiota, leading to dysbiosis, marked by reduced diversity and increased numbers of harmful bacteria. This imbalance contributes to intestinal inflammation and impaired nutrient absorption. Given the close interdependence between mucus properties, biofilm formation, and microbiota composition, future therapeutic strategies that simultaneously target these factors may further enhance gastrointestinal health in individuals with CF. A comprehensive understanding of gut microbiome dynamics in response to CFTR protein modulators is essential to elucidate the broader benefits of these therapies. Such insight underscores the importance of integrating microbiome-focused interventions into comprehensive CF care.

## Figures and Tables

**Table 1:** Gut microbiota differences between healthy individuals and CF patients.

Category	Healthy People	People with Cystic Fibrosis	References
Dominant bacteria	*Bifidobacterium*, *Lactobacillus*, *Faecalibacterium*, *Roseburia*, and *Bacteroides*	*E. coli*, *S. aureus*, *P. aeruginosa*, and *C. difficile*	[1,2,3,4,7,14,16,21,22,23]
Microbiome diversity	High: Enables symbiotic interactions and stabilises the intestinal environment	Low: Leads to dysbiosis and excessive growth of pathogens	[2,3,4,7,8,9,20]
SCFA production	High: Supports anti-inflammatory processes, regulates metabolism	Reduced: Low SCFA leads to inflammation	[4,10,11,12,13,16]
Effect on absorption	Increased absorption of minerals and vitamins; stabilisation of metabolism	Impaired absorption of nutrients due to inflammation and pathogens	[2,3,4,6,7]
Inflammation	Low: Beneficial microflora inhibits inflammation	High: The presence of pathogens leads to chronic inflammation	[2,3,4,7,8,20]
Bacterial interactions	Symbiosis between bacteria: Co-production of metabolites	Antagonism: Pathogenic bacteria dominate and compete with beneficial bacteria	[2,4,7,8,9,17]
Weight to achieve	It enables healthy development and weight maintenance	This often leads to malnutrition and weight loss	[2,4,6,7]
Toxin production	Low: No pathogenic strains	High: Presence of toxin-secreting pathogens	[2,3,4,7,22,23]

## References

[1] López-Valdez J.A., Aguilar-Alonso L.A., Gándara-Quezada V., Ruiz-Rico G.E., Ávila-Soledad J.M., Reyes A.A., Pedroza-Jiménez F.D. (2021). Cystic fibrosis: Current concepts. Boletín Médico Hosp. Infant. México.

[2] Mall M.A., Burgel P.R., Castellani C., Davies J.C., Salathe M., Taylor-Cousar J.L. (2024). Cystic fibrosis. Nat. Rev. Dis. Primers.

[3] Ooi C.Y., Durie P.R. (2016). Cystic fibrosis from the gastroenterologist’s perspective. Nat. Rev. Gastroenterol. Hepatol..

[4] Tam R.Y., van Dorst J.M., McKay I., Coffey M., Ooi C.Y. (2022). Intestinal Inflammation and Alterations in the Gut Microbiota in Cystic Fibrosis: A Review of the Current Evidence, Pathophysiology and Future Directions. J. Clin. Med..

[5] Jones E.J., Booth C., Fonseca S., Parker A., Cross K., Miquel-Clopés A., Hautefort I., Mayer U., Wileman T., Stentz R. (2020). The Uptake, Trafficking, and Biodistribution of Bacteroides thetaiotaomicron Generated Outer Membrane Vesicles. Front. Microbiol..

[6] Turck D., Braegger C.P., Colombo C., Declercq D., Morton A., Pancheva R., Robberecht E., Stern M., Strandvik B., Wolfe S. (2016). ESPEN-ESPGHAN-ECFS guidelines on nutrition care for infants, children, and adults with cystic fibrosis. Clin. Nutr..

[7] De Freitas M.B., Moreira E.A.M., Tomio C., Moreno Y.M.F., Daltoe F.P., Barbosa E., Ludwig Neto N., Buccigrossi V., Guarino A. (2018). Altered intestinal microbiota composition, antibiotic therapy and intestinal inflammation in children and adolescents with cystic fibrosis. PLoS ONE.

[8] Afzaal M., Saeed F., Shah Y.A., Hussain M., Rabail R., Socol C.T., Hassoun A., Pateiro M., Lorenzo J.M., Rusu A.V. (2022). Human gut microbiota in health and disease: Unveiling the relationship. Front. Microbiol..

[9] Hou K., Wu Z.X., Chen X.Y., Wang J.-Q., Zhang D., Xiao C., Zhu D., Koya J.B., Wei L., Li J. (2022). Microbiota in health and diseases. Signal Transduct. Target. Ther..

[10] Van-Wehle T., Vital M. (2024). Investigating the response of the butyrate production potential to major fibers in dietary intervention studies. NPJ Biofilms Microbiomes.

[11] Mansuy-Aubert V., Ravussin Y. (2023). Short chain fatty acids: The messengers from down below. Front. Neurosci..

[12] Zhang D., Jian Y.P., Zhang Y.N., Li Y., Gu L.-T., Sun H.-H., Liu M.-D., Zhou H.-L., Wang Y.-S., Xu Z.-X. (2023). Short-chain fatty acids in diseases. Cell Commun. Signal..

[13] Recharla N., Geesala R., Shi X.-Z. (2023). Gut Microbial Metabolite Butyrate and Its Therapeutic Role in Inflammatory Bowel Disease: A Literature Review. Nutrients.

[14] Zhang C., Ma K., Nie K., Deng M., Luo W., Wu X., Huang Y., Wang X. (2022). Assessment of the safety and probiotic properties of Roseburia intestinalis: A potential “Next Generation Probiotic”. Front. Microbiol..

[15] De Jesus L.C.L., Aburjaile F.F., De Jesus Sousa T., Felice A.G., De Castro Soares S., Alcantara L.C.J., De Carvalho Azevedo V.A. (2022). Genomic Characterization of *Lactobacillus delbrueckii* Strains with Probiotics Properties. Front. Bioinform..

[16] Liu X.-F., Shao J.-H., Liao Y.-T., Wang L.-N., Jia Y., Dong P.-J., Liu Z.-Z., He D.-D., Li C., Zhang X. (2023). Regulation of short-chain fatty acids in the immune system. Front. Immunol..

[17] Xiao M., Zhang C., Duan H., Narbad A., Zhao J., Chen W., Zhai Q., Yu L., Tian F. (2024). Cross-feeding of bifidobacteria promotes intestinal homeostasis: A lifelong perspective on the host health. NPJ Biofilms Microbiomes.

[18] Ramakrishna C., Kujawski M., Chu H., Li L., Mazmanian S.K., Cantin E.M. (2019). Bacteroides fragilis polysaccharide A induces IL-10 secreting B and T cells that prevent viral encephalitis. Nat. Commun..

[19] Durant L., Stentz R., Noble A., Brooks J., Gicheva N., Reddi D., O’Connor M.J., Hoyles L., McCartney A.L., Man R. (2020). Bacteroides thetaiotaomicron-derived outer membrane vesicles promote regulatory dendritic cell responses in health but not in inflammatory bowel disease. Microbiome.

[20] Wrigley-Carr H.E., van Dorst J.M., Ooi C.Y. (2022). Intestinal dysbiosis and inflammation in cystic fibrosis impacts gut and multi-organ axes. Med. Microecol..

[21] Doranga S., Krogfelt K.A., Cohen P.S., Conway T. (2024). Nutrition of *Escherichia coli* within the intestinal microbiome. EcoSal Plus.

[22] Gomes T.A., Elias W.P., Scaletsky I.C., Guth B.E., Rodrigues J.F., Piazza R.M., Ferreira L.C., Martinez M.B. (2016). Diarrheagenic Escherichia coli. Braz. J. Microbiol..

[23] Lorusso A.B., Carrara J.A., Barroso C.D.N., Tuon F.F., Faoro H. (2022). Role of Efflux Pumps on Antimicrobial Resistance in Pseudomonas aeruginosa. Int. J. Mol. Sci..

[24] Krysenko S., Wohlleben W. (2022). Polyamine and Ethanolamine Metabolism in Bacteria as an Important Component of Nitrogen Assimilation for Survival and Pathogenicity. Med. Sci..

[25] Lundgren B.R., Sarwar Z., Pinto A., Ganley J.G., Nomura C.T. (2016). Ethanolamine Catabolism in Pseudomonas aeruginosa PAO1 Is Regulated by the Enhancer-Binding Protein EatR (PA4021) and the Alternative Sigma Factor RpoN. J. Bacteriol..

[26] Kaval K.G., Garsin D.A. (2018). Ethanolamine Utilization in Bacteria. mBio.

[27] Reese A.T., Pereira F.C., Schintlmeister A., Berry D., Wagner M., Hale L.P., Wu A., Jiang S., Durand H.K., Zhou X. (2018). Microbial nitrogen limitation in the mammalian large intestine. Nat. Microbiol..

[28] Hammer N.D., Skaar E.P. (2011). Molecular mechanisms of Staphylococcus aureus iron acquisition. Annu. Rev. Microbiol..

[29] Ghssein G., Ezzeddine Z. (2022). A Review of *Pseudomonas aeruginosa* Metallophores: Pyoverdine, Pyochelin and Pseudopaline. Biology.

[30] Alford M.A., Mann S., Akhoundsadegh N., Hancock R.E.W. (2022). Competition between *Pseudomonas aeruginosa* and *Staphylococcus aureus* is dependent on intercellular signaling and regulated by the NtrBC two-component system. Sci. Rep..

[31] Alam M.Z., Madan R. (2024). *Clostridioides difficile* Toxins: Host Cell Interactions and Their Role in Disease Pathogenesis. Toxins.

[32] Camus L., Briaud P., Bastien S., Elsen S., Doléans-Jordheim A., Vandenesch F., Moreau K. (2020). Trophic cooperation promotes bacterial survival of Staphylococcus aureus and Pseudomonas aeruginosa. ISME J..

[33] Pensinger D.A., Dobrila H.A., Stevenson D.M., Hryckowian N.D., Amador-Noguez D., Hryckowian A.J. (2024). Exogenous butyrate inhibits butyrogenic metabolism and alters virulence phenotypes in *Clostridioides difficile*. mBio.

[34] Baldassare M.A., Bhattacharjee D., Coles J.D., Nelson S., McCollum C.A., Seekatz A.M. (2023). Butyrate enhances Clostridioides difficile sporulation in vitro. J. Bacteriol..

[35] Gregory A.L., Pensinger D.A., Hryckowian A.J. (2021). A short chain fatty acid-centric view of Clostridioides difficile pathogenesis. PLoS Pathog..

[36] Sheng Y.H., Hasnain S.Z. (2022). Mucus and Mucins: The Underappreciated Host Defence System. Front. Cell. Infect. Microbiol..

[37] Suriano F., Nyström E.E.L., Sergi D., Gustafsson J.K. (2022). Diet, microbiota, and the mucus layer: The guardians of our health. Front. Immunol..

[38] Damianos J., Abdelnaem N., Camilleri M. (2025). Gut Goo: Physiology, Diet, and Therapy of Intestinal Mucus and Biofilms in Gastrointestinal Health and Disease. Clin. Gastroenterol. Hepatol..

[39] Joja M., Grant E.T., Desai M.S. (2025). Living on the edge: Mucus-associated microbes in the colon. Mucosal Immunol..

[40] Elzinga J., Narimatsu Y., de Haan N., Clausen H., de Vos W.M., Tytgat H.L.P. (2024). Binding of *Akkermansia muciniphila* to mucin is *O*-glycan specific. Nat. Commun..

[41] Engevik M.A., Luk B., Chang-Graham A.L., Hall A., Herrmann B., Ruan W., Endres B.T., Shi Z., Garey K.W., Hyser J.M. (2019). *Bifidobacterium dentium* Fortifies the Intestinal Mucus Layer via Autophagy and Calcium Signaling Pathways. mBio.

[42] Chen See J.R., Leister J., Wright J.R., Kruse P.I., Khedekar M.V., Besch C.E., Kumamoto C.A., Madden G.R., Stewart D.B., Lamendella R. (2024). *Clostridioides difficile* infection is associated with differences in transcriptionally active microbial communities. Front. Microbiol..

[43] Cheng Y., Cai D., Zheng Y., Yan S., Wu L., Li C., Song W., Xin T., Lv S., Huang R. (2020). Microscopic Mechanism of Carbon-Dopant Manipulating Device Performance in CGeSbTe-Based Phase Change Random Access Memory. ACS Appl. Mater. Interfaces.

[44] Fekete E., Buret A.G. (2023). The role of mucin *O*-glycans in microbiota dysbiosis, intestinal homeostasis, and host-pathogen interactions. Am. J. Physiol.-Gastrointest. Liver Physiol..

[45] Valiei A., Dickson A., Aminian-Dehkordi J., Mofrad M.R.K. (2024). Metabolic interactions shape emergent biofilm structures in a conceptual model of gut mucosal bacterial communities. NPJ Biofilms Microbiomes.

[46] Liu H.Y., Prentice E.L., Webber M.A. (2024). Mechanisms of antimicrobial resistance in biofilms. NPJ Antimicrob. Resist..

[47] Jandl B., Dighe S., Baumgartner M., Makristathis A., Gasche C., Muttenthaler M. (2024). Gastrointestinal Biofilms: Endoscopic Detection, Disease Relevance, and Therapeutic Strategies. Gastroenterology.

[48] Ghannoum M., Ghannoum A., Hager C., Retuerto M., Isham N., McCormick T.S. (2019). The Probiotic BIOHM Improves Nutrient Absorption by Disrupting Gastrointestinal Biofilms. J. Probiotics Health.

[49] Molobela I., Cloete T., Beukes M. (2010). Protease and amylase enzymes for biofilm removal and degradation of extracellular polymeric substances (EPS) produced by Pseudomonas fluorescens bacteria. Afr. J. Microbiol. Res..

[50] Tielen P., Kuhn H., Rosenau F., Jaeger K.-E., Flemming H.-C., Wingender J. (2013). Interaction between extracellular lipase LipA and the polysaccharide alginate of *Pseudomonas aeruginosa*. BMC Microbiol..

[51] Ruch T.R., Engel J.N. (2017). Targeting the Mucosal Barrier: How Pathogens Modulate the Cellular Polarity Network. Cold Spring Harb. Perspect. Biol..

[52] Amati F., Leonardi G., Contarini M., Morlacchi L.C., Stainer A., Pizzamiglio G., Aliberti S., Blasi F., Gramegna A. (2024). Immunodeficiencies and CFTR dysfunction: Results from a systematic screening in a cohort of adults with cystic fibrosis and CFTR-related disorders. Ther. Adv. Respir. Dis..

[53] Purushothaman A.K., Nelson E.J.R. (2023). Role of innate immunity and systemic inflammation in cystic fibrosis disease progression. Heliyon.

[54] Kristensen M., Prevaes S.M.P.J., Kalkman G., Tramper-Stranders G.A., Hasrat R., de Winter-de Groot K.M., Janssens H.M., Tiddens H.A., van Westreenen M., Sanders E.A.M. (2020). Development of the gut microbiota in early life: The impact of cystic fibrosis and antibiotic treatment. J. Cyst. Fibros..

[55] Mazziotta C., Tognon M., Martini F., Torreggiani E., Rotondo J.C. (2023). Probiotics Mechanism of Action on Immune Cells and Beneficial Effects on Human Health. Cells.

[56] Ney L.M., Wipplinger M., Grossmann M., Engert N., Wegner V.D., Mosig A.S. (2023). Short chain fatty acids: Key regulators of the local and systemic immune response in inflammatory diseases and infections. Open Biol..

[57] Baldwin-Hunter B.L., Rozenberg F.D., Annavajhala M.K., Park H., DiMango E.A., Keating C.L., Uhlemann A.C., Abrams J.A. (2023). The gut microbiome, short chain fatty acids, and related metabolites in cystic fibrosis patients with and without colonic adenomas. J. Cyst. Fibros..

[58] Overby H.B., Ferguson J.F. (2021). Gut Microbiota-Derived Short-Chain Fatty Acids Facilitate Microbiota: Host Cross talk and Modulate Obesity and Hypertension. Curr. Hypertens. Rep..

[59] Borisova D., Paunova-Krasteva T., Strateva T., Stoitsova S. (2025). Biofilm Formation of Pseudomonas aeruginosa in Cystic Fibrosis: Mechanisms of Persistence, Adaptation, and Pathogenesis. Microorganisms.

[60] Jean-Pierre V., Boudet A., Sorlin P., Menetrey Q., Chiron R., Lavigne J.P., Marchandin H. (2022). Biofilm Formation by *Staphylococcus aureus* in the Specific Context of Cystic Fibrosis. Int. J. Mol. Sci..

[61] Uruén C., Chopo-Escuin G., Tommassen J., Mainar-Jaime R.C., Arenas J. (2020). Biofilms as Promoters of Bacterial Antibiotic Resistance and Tolerance. Antibiotics.

[62] Thavamani A., Salem I., Sferra T.J., Sankararaman S. (2021). Impact of Altered Gut Microbiota and Its Metabolites in Cystic Fibrosis. Metabolites.

[63] Liu Q., Tian X., Maruyama D., Arjomandi M., Prakash A. (2021). Lung immune tone via gut-lung axis: Gut-derived LPS and short-chain fatty acids’ immunometabolic regulation of lung IL-1β, FFAR2, and FFAR3 expression. Am. J. Physiol. Lung Cell. Mol. Physiol..

[64] Kelly C.J., Zheng L., Campbell E.L., Saeedi B., Scholz C.C., Bayless A.J., Wilson K.E., Glover L.E., Kominsky D.J., Magnuson A. (2015). Crosstalk between Microbiota-Derived Short-Chain Fatty Acids and Intestinal Epithelial HIF Augments Tissue Barrier Function. Cell Host Microbe.

[65] Smith P.M., Howitt M.R., Panikov N., Michaud M., Gallini C.A., Bohlooly-Y M., Glickman J.N., Garrett W.S. (2013). The microbial metabolites, short-chain fatty acids, regulate colonic T_reg_ cell homeostasis. Science.

[66] Caley L.R., White H., de Goffau M.C., Floto R.A., Parkhill J., Marsland B., Peckham D.G. (2023). Cystic Fibrosis-Related Gut Dysbiosis: A Systematic Review. Dig. Dis. Sci..

[67] Suppakitjanusant P., Wang Y., Sivapiromrat A.K., Hu C., Binongo J., Hunt W.R., Weinstein S., Jathal I., Alvarez J.A., Chassaing B. (2024). Impact of high-dose cholecalciferol (vitamin D3) and inulin prebiotic on intestinal and airway microbiota in adults with cystic fibrosis: A 2 × 2 randomized, placebo-controlled, double-blind pilot study. J. Clin. Transl. Endocrinol..

[68] Caverly L.J., Riquelme S.A., Hisert K.B. (2022). The Impact of Highly Effective Modulator Therapy on Cystic Fibrosis Microbiology and Inflammation. Clin. Chest Med..

[69] Marsh R., Santos C.D., Yule A., Dellschaft N.S., Hoad C.L., Ng C., Major G., Smyth A.R., Rivett D., van der Gast C. (2024). Impact of extended Elexacaftor/Tezacaftor/Ivacaftor therapy on the gut microbiome in cystic fibrosis. J. Cyst. Fibros..

[70] Pope C.E., Vo A.T., Hayden H.S., Weiss E.J., Durfey S., McNamara S., Ratjen A., Grogan B., Carter S., Nay L. (2021). Changes in fecal microbiota with CFTR modulator therapy: A pilot study. J. Cyst. Fibros..

[71] Lee J.-A., Cho A., Huang E., Xu Y., Quach H., Hu W.J., Wong A. (2021). Gene therapy for cystic fibrosis: New tools for precision medicine. J. Transl. Med..

